# Smart-Sleeve: A Wearable Textile Pressure Sensor Array for Human Activity Recognition

**DOI:** 10.3390/s22051702

**Published:** 2022-02-22

**Authors:** Guanghua Xu, Quan Wan, Wenwu Deng, Tao Guo, Jingyuan Cheng

**Affiliations:** 1 School of Data Science, University of Science and Technology of China, Hefei 230026, China; xgh@mail.ustc.edu.cn; 2 School of Computer Science and Technology, University of Science and Technology of China, Hefei 230026, China; wanquan2020@mail.ustc.edu.cn (Q.W.); dengwenw@mail.ustc.edu.cn (W.D.); gtpplab@mail.ustc.edu.cn (T.G.)

**Keywords:** human activity recognition, textile pressure matrix, smart sleeves

## Abstract

Human activity recognition is becoming increasingly important. As contact with oneself and the environment accompanies almost all human activities, a Smart-Sleeve, made of soft and stretchable textile pressure sensor matrix, is proposed to sense human contact with the surroundings and identify performed activities in this work. Additionally, a dataset including 18 activities, performed by 14 subjects in 10 repetitions, is generated. The Smart-Sleeve is evaluated over six classical machine learning classifiers (support vector machine, k-nearest neighbor, logistic regression, random forest, decision tree and naive Bayes) and a convolutional neural network model. For classical machine learning, a new normalization approach is proposed to overcome signal differences caused by different body sizes and statistical, geometric, and symmetry features are used. All classification techniques are compared in terms of classification accuracy, precision, recall, and F-measure. Average accuracies of 82.02% (support vector machine) and 82.30% (convolutional neural network) can be achieved in 10-fold cross-validation, and 72.66% (support vector machine) and 74.84% (convolutional neural network) in leave-one-subject-out validation, which shows that the Smart-Sleeve and the proposed data processing method are suitable for human activity recognition.

## 1. Introduction

Human activity recognition (HAR) is an important task in pervasive computing [1] and computer vision [2]. While inertial-based and vision-based sensings are enjoying rapid growth in recent years, mainly thanks to the development of hardware and algorithms, new aspects such as contact are still to be explored. Pirsiavash et al. [3] suggest that real-world activities of daily living recognition are “all about the objects”, and, in particular, “all about the objects being interacted with”. We notice that almost all human activities are accompanied by contact with oneself or the external environment, which might contain useful information for human activity recognition. For human beings, the hands are most frequently used for contact interactions. While a pressure sensing device directly on the hand might be impractical (e.g., gloves may affect the sense of touch, and it may be rude to shake hands with gloves in some social situations), we turn our attention to the arm and propose a Smart-Sleeve consisting of a matrix of textile-based pressure sensors for HAR.

The main contributions of this work are as follows:

1. A Smart-Sleeve based on pressure mapping textiles is proposed for HAR. The sleeve is soft, stretchable, washable, and can be easily incorporated into ordinary clothing.
2. Both classic machine learning and deep learning methods are used to evaluate the performance of the Smart-Sleeve for everyday activity recognition. To normalize the data differences caused by different body sizes, a new preprocessing method is adopted. A feature set of 100 features, including statistical, geometric, and symmetry features, is proposed.
3. Our dataset is open to the public, including 18 daily activities performed by 14 participants in an office scenario. To the best of our knowledge, there are no publicly available datasets of pressure sensor-based sleeves for HAR.

The rest of the paper is organized as follows: literature study is discussed in Section 2, systematic design and dataset description are explained in Section 3, data processing methods are presented in Section 4, experimental evaluations are described in Section 5, the paper is concluded in Section 6 and discussed in Section 7.

## 2. Related Work

### 2.1. Systems for Human Activity Recognition

HAR systems can usually be divided into two main categories: environmental and wearable.

Environmental sensors are usually hidden in the surroundings, such as cameras [4] and wireless signaling devices [5,6,7], which are usually not easily detectable and do not interfere with the normal activities of the user. Particularly, visual sensor-based systems are widely used [2,8] and have achieved good results. However, they also face challenges in terms of personal privacy and spatial constraints, such as cameras being placed in public places, capturing data of the subject while also directly accessing data from other unrelated people. Pressure sensors have also been used in the format of environmental systems, as most daily human activities, e.g., locomotion, exercises, and resting, are heavily synchronized with the tactile interactions between the human and the environment. For example, Luo et al. [9] propose a 3D human pose estimation approach using the pressure maps recorded by a tactile carpet as input. Casas et al. [10] estimate the patient’s posture from pressure sensors’ data mapped to images.

Wearable systems are always with the user, and not limited to certain locations such as the environmental systems. Pirsiavash et al. [3] use the first-person camera to detect activities of daily living, but there are significant challenges such as limitations of currently available battery technology. The rapid development of microelectromechanical systems technology has contributed to the development of low-power, low-cost, small-size, andlightweight inertial sensors [11,12,13], although recently there has been progress in arm motion tracking [14] and dynamic motion capture of human lower limbs [15] using a single inertial measurement unit (IMU) sensor. The number and the placement of inertial sensors on the human body have a direct impact on activity recognition, in terms of the variety of activities to monitor and the precision of their classification [16,17,18]. The use of many IMU sensors not only limits the deployment phase but also increases the difficulty and discomfort for users.

Some representative HAR systems are listed in Table 1. Although existing HAR systems, such as cameras and IMUs, have achieved a good accuracy, the number of researchers starting to pay attention to textile sensors and their applications is growing due to their soft, deformable, and stretchable characteristics. Table 2 lists the advantages and disadvantages of some commonly used sensors for HAR.

### 2.2. Textile Sensors and Applications

As early as 1996, Inaba et al. [21] presented a tactile system that covers the entire body of a robot with textile sensors. Since then, textile sensors have received more and more attention from researchers.

Textile sensors can be easily integrated into the environment and unobtrusively monitor daily life. Sundholm et al. [19] present a textile pressure sensor matrix integrated into a gym mat for monitoring strength-related exercises that are performed on the ground. Vega-barbas et al. [22] present a smart toy for the assessment of psychomotor development in early childhood. Xu et al. [23] propose a smart cushion based on a textile pressure sensor array to monitor sitting postures. Similarly, some pressure-sensitive bedsheet systems are designed for sleep posture monitoring [24,25]. However, they, similar to other environmental sensors, are limited by certain locations.

Textile sensors have been integrated into clothing owing to their soft, deformable, and stretchable characteristics. Liu et al. [26] focus on the primary use of conductive stretchable fabrics to monitor joint angular motion. Voit et al. [27] explore how the arm posture of the user can be detected with a smart fabric and can be used as input. Both of them, however, do not take into account the contact information with other limbs and the external environment.

Recently, advances in wearable textile pressure sensors have benefited applications based on the contact information in a variety of contexts. Google proposes Project Jacquard [28], which allows adding interactivity to smart clothing invisibly and unobtrusively, without compromising the look and feel of clothes. Microsoft also proposes Project Tasca [29], a pocket-based textile sensor that detects users’ input and recognizes everyday objects that a user carries in the pockets of a pair of pants. Parzer et al. present SmartSleeve, a deformable textile sensor, which can sense both surface and deformation gestures in real-time [20,30]. Leong et al. [31] present a prototype of a prosthetic-sensing wearable for the sensory augmentation of lower-limb prosthetics. They all focus on enhancing the interactivity or sensing capabilities of clothing, while there are few efforts to identify and analyze the daily activities of the wearer.

In summary, to the best of our knowledge, there are few studies about textile pressure-sensing sleeves for HAR, and no relevant datasets are available. Therefore, in this work, a smart sleeve is designed and its ability to recognize daily activities is evaluated.

## 3. System and Experiment Design

### 3.1. Smart-Sleeve

**Figure 1:**
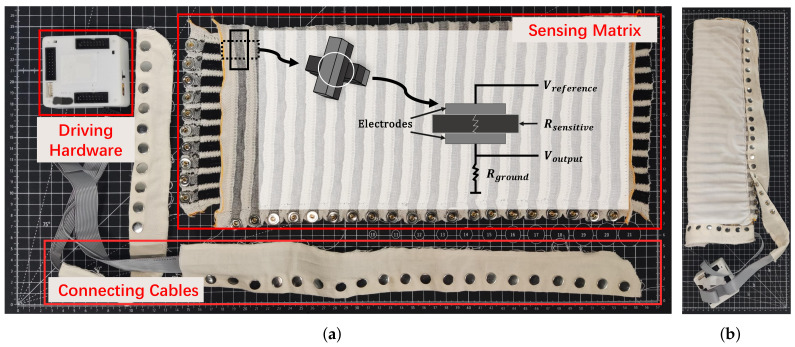
Smart-Sleeve. (**a**) The textile matrix. (**b**) The cable and driving hardware can be easily removed from the textile part.

Based on the previous work [30,32], the Smart-Sleeve is designed using double-sided weft-knitted fabrics (shown in Figure 1). The gray stripes are made of metallic conductive yarns and the black stripes are made of polymer yarns with carbon powder mixed in, serving as the sensitive layer. The top fabric contains 20 stripes, and the bottom one has 10 stripes. Orthogonally stacked together, they form a matrix with 200 sensing points. The size is 40 cm × 18 cm. To prevent short circuits caused by adjacent sensors touching in a bent arm posture, it is covered with a layer of flexible skin-friendly insulating fabric. The matrix is driven and scanned by a hardware mainly consisting of a STM32F303ZET6 microcontroller and a Bluetooth 4.0 module. Data are sampled by 12-bit ADCs at 50 Hz and transmitted to a smartphone via Bluetooth. Experiments show that this hardware with a 2200 mAh battery could carry out more than ten hours of continuous data acquisition and transmission. A CSV file is used to save the data and is later transferred to the computer for data processing. Data from all 200 points in a matrix format sampled at the same time are called a “frame” or “pressure image” and the size is 20×10 .

### 3.2. Experiment Design

**Figure 2:**
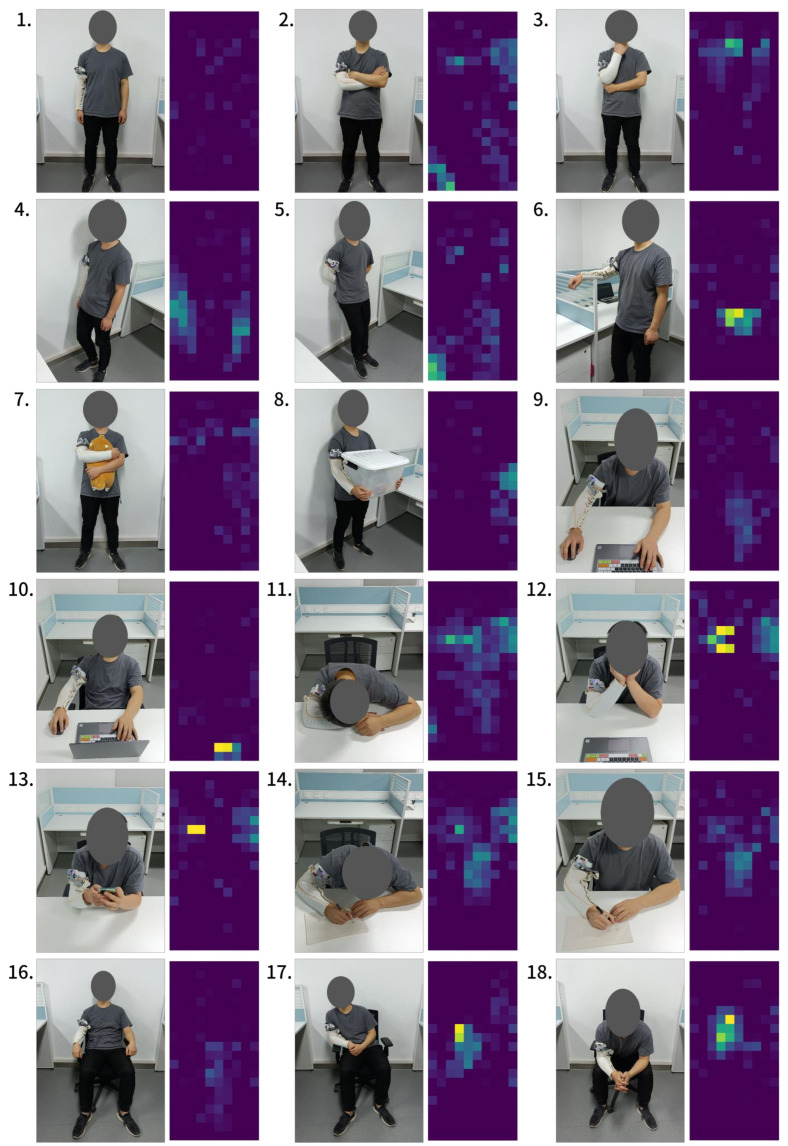
Eighteen activities and the corresponding pressure distribution on the Smart-Sleeve. (**1**) Stand. (**2**) Fold arms. (**3**) Think. (**4**) Side against the wall. (**5**) Back against the wall. (**6**) Arm on the baffle. (**7**) Hug a doll. (**8**) Carry a box. (**9**) Lean forward at work. (**10**) Lean back at work. (**11**) Sleep on the table. (**12**) Hold cheeks. (**13**) Play mobile phone. (**14**) Write with a hunchback. (**15**) Write with a straight back. (**16**) Sit with hands on the armrests. (**17**) Sit leaning to the right. (**18**) Sit with arms on the legs.

To evaluate the Smart-Sleeve’s ability in recognizing daily activities, 18 common activities were selected, as shown in Figure 2, and 14 healthy subjects (3 females) were invited to participate in the experiment and their related body information were collected, as shown in Table 3. The participants are all right-handed and therefore wore the sleeve on their right arm. The experiment was divided into 10 rounds; in each round the 18 activities were placed into random order and repeated once. To simulate everyday usage, the Smart-Sleeve was taken off and reworn after each round.

Most of these activities were in a stationary posture. It was also observed that the pressure images do not change significantly during dynamic activities such as playing with the phone and writing. Therefore, in each activity, a sample containing an “average image” by calculating the average of pixels at the same position of all images was obtained. Finally, a dataset consisting of 2520 samples (14 subjects, 10 rounds per subject, 18 actions per round) was generated.

## 4. Data Processing

### 4.1. Preprocessing

**Figure 3:**
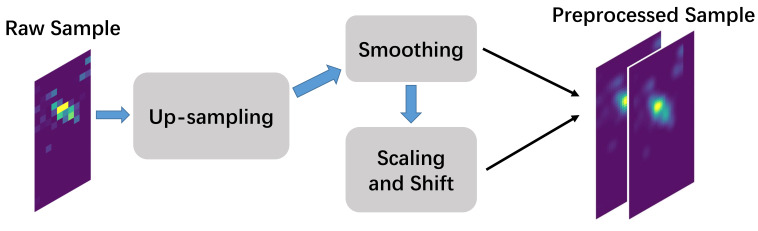
The preprocessing workflow. The number of pressure images is doubled.

The samples are first preprocessed to enhance the signal quality and remove the influence of the body difference, as shown in Figure 3.

#### 4.1.1. Upsampling and Smoothing

**Figure 4:**
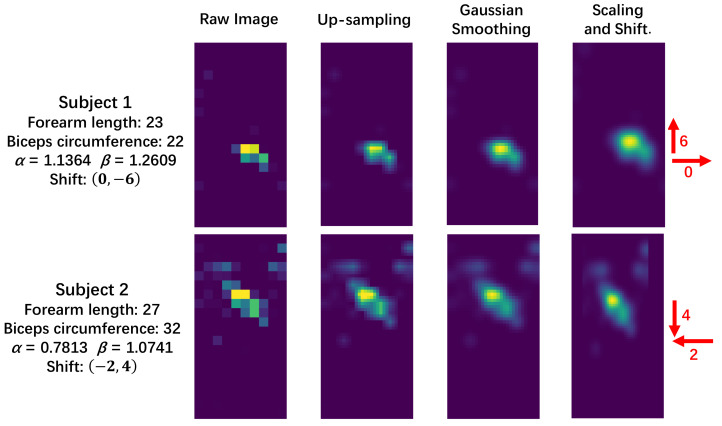
Data preprocessing for different subjects in activity 6 (arm on the baffle).

Previous work [19] shows that upsampling using bilinear interpolation creates better images. Not only is the pressure image visually smoother, but its classification result also improves. We thus upsample every pressure image by 3, then smooth it with a 5×5 Gaussian filter. The visual effect is shown in Figure 4.

#### 4.1.2. Scaling and Shift

In contact with the external environment, such as baffles and tables, thin subjects produce less contact area, as shown in Figure 4. As body size varies considerably among the subjects, shown in Table 3, scaling the pressure image based on anthropometric information may improve classification accuracy. The parameters α(i) and β(i) are used to represent the row and column scale ratios of the *i*-th subject, as given in Equations (1) and (2).

(1) $$\alpha \left(i\right)=\frac{\sum_{k=1}^{n}L_{FA}\left(k\right)}{n}\times \frac{1}{L_{FA}\left(i\right)}$$

(2) $$\beta \left(i\right)=\frac{\sum_{k=1}^{n}L_{BC}\left(k\right)}{n}\times \frac{1}{L_{BC}\left(i\right)}$$

where *n* is the number of subjects, $L_{FA}\left(i\right)$ denotes the forearm length of the *i*-th subject, and $L_{BC}\left(i\right)$ denotes the biceps circumference of the *i*-th subject. After scaling using bilinear interpolation, the pressure images from tall and fat subjects become smaller and counterwise for thin and small subjects.

Because in normal life clothes are put on and taken off every day, the subjects were instructed to rewear the Smart-Sleeve after each round. Offsets thus exist in each round. To remove this effect, we first combine all the pressure images in the same round into one “exposure image” (the image obtained by summing the pixels at the same position of all images), and calculate the center of mass coordinates of each “exposure image”. Assuming that these coordinates should be the same in the absence of offsets, the offset for the pressure images of each round is obtained after using the mean of these coordinates as the target coordinate. Based on this offset, we perform the same shift operation on all scaled frames within each round. Data out of the normal region are cropped. To minimize missing image information, the edges of the images are zero-filled at the beginning of this step, and the final image size is 84×42 , which is larger than the smoothed one (the size of the raw image is 20×10 , then rises to 60×30 after upsampling by 3). The scaled and shifted pressure image is shown in Figure 4. Because scaling and shifting might discard some useful information from the original image, the new image is added to the sample instead of overwriting the original image.

### 4.2. Feature Extraction

Guo et al. [33] propose a feature library including 1830 ready-to-use features based on the work of Zhou et al. [34], which contains 38 spatial features and 23 frame descriptors. Liu et al. [25] define 32 geometric features for pressure sensor-based smart sheet data, most of which are related to the location of human body parts. In this work, we organize the previous work [19,25,33,34,35,36], supplement more features, and divide them into three categories: the statistical, the geometric, and the symmetry. Our static feature set contains 100 features considering only a single pressure image. As each instance is represented by two pressure frames, 200 features for each instance are obtained.

#### 4.2.1. Statistical Features

For all pixels on the pressure image, the following eight statistical features are calculated:

$Feat_{1}$
 to 
$Feat_{4}$
:Maximum, median, sum, and range (maximum–median).
$Feat_{5}$
 to 
$Feat_{8}$
:Average, variance, mean absolute deviation, and entropy of all pixel values in the pressure image, as defined in [34].

#### 4.2.2. Geometric Features

**Figure 5:**
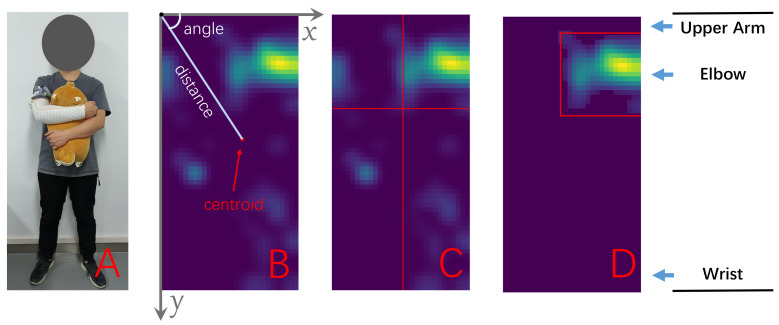
Geometric features calculation. (**A**) Activity 7 (hug a doll), corresponding to the pressure image. (**B**) The coordinates ( $Feat_{9}$ , $Feat_{10}$ ), the distance from the origin ( $Feat_{13}$ ), and the angle ( $Feat_{15}$ ) of the centroid. (**C**) Coverage by regions ( $Feat_{33}$ to $Feat_{36}$ ). (**D**) The masked image and the bounding rectangle.

Using the image’s upper left corner as the origin, its short side as the *x*-axis, and the long side as the *y*-axis, the coordinate system is defined, shown in Figure 5B. The following geometric features are extracted.



$Feat_{9}$
, 
$Feat_{10}$
:The centroid coordinate *x* and *y*.
$Feat_{11}$
, 
$Feat_{12}$
:The centre of mass coordinate *x* and *y*.
$Feat_{13}$
:The distance from the centroid to the origin.
$Feat_{14}$
:The distance from the centre of mass to the origin.
$Feat_{15}$
:The angle between the line from the origin to the centroid and the positive direction of the *x*-axis.
$Feat_{16}$
:The angle between the line from the origin to the centre of mass and the positive direction of the *x*-axis.
$Feat_{17}$
 to 
$Feat_{20}$
:Width, height, aspect ratio, and area of the bounding rectangle of the pressure image.
$Feat_{21}$
:Area (the number of pixels after thresholding with a value of 2).
$Feat_{22}$
 to 
$Feat_{28}$
:Hu’s seven invariant moments [37], which are rotation, translation, and scale invariant.
$Feat_{29}$
:Coverage (proportion of image covered).
$Feat_{30}$
 to 
$Feat_{32}$
:The coverage for the pixels that contain 25%, 50%, and 75% of the total pressure.
$Feat_{33}$
 to 
$Feat_{36}$
:The coverage over four fixed rectangular regions.
$Feat_{37}$
:The number of contours.
$Feat_{38}$
:Area of the contour containing the largest area.
$Feat_{39}$
:Pressure of the contour containing the largest pressure.
$Feat_{40}$
:Intensity of pressure of the contour containing the largest intensity of pressure.
$Feat_{41}$
 to 
$Feat_{52}$
:
$Feat_{9}$
 to 
$Feat_{20}$
 of the masked image.

Liu et al. [25] define the coverage which is “the number of pixels that have non-negative sensor values divided by the total number of pixels”. The coverages ( $Feat_{29}$ to $Feat_{32}$ ) are calculated, and for the Smart-Sleeve, the pressure image is divided into four regions using the upper third point of the long side and the center point of the short side, as shown in Figure 5C. We calculate the coverage by regions ( $Feat_{33}$ to $Feat_{36}$ ).

The contours of the pressure image also contain a lot of information. Using the mean value of all pixels in the image as the threshold, we binarize the pressure image and obtain the contours. The number of the contours is counted as $Feat_{37}$ . For each contour, area (number of pixels), pressure (sum of pixels), and intensity of pressure (pressure divided by area) of the contained area are calculated, and we take the maximum value of each of them as $Feat_{38}$ , $Feat_{39}$ , and $Feat_{40}$ . By leaving only the pixels surrounded by the contour with maximum pressure, a masked image is obtained, as in Figure 5D, and then is used to calculate $Feat_{9}$ to $Feat_{20}$ again as $Feat_{41}$ to $Feat_{52}$ .

#### 4.2.3. Symmetry Features

The pressure image is divided into the left and the right parts by the x-coordinate of the center of mass, and the following features are extracted.

$Feat_{53}$
, 
$Feat_{54}$
:The area of each side.
$Feat_{55}$
, 
$Feat_{56}$
:the pressure of each side.
$Feat_{57}$
:The ratio of area of both sides.
$Feat_{58}$
:The ratio of pressure of both sides.

Similarly, the y-coordinate of the center of mass is used to divide the pressure image into the upper and the lower parts, and $Feat_{53}$ to $Feat_{58}$ is calculated again as $Feat_{59}$ to $Feat_{64}$ . For the centroid, the same steps are performed and $Feat_{65}$ to $Feat_{76}$ are obtained. For the masked image mentioned above, we also calculate its symmetry features $Feat_{77}$ to $Feat_{100}$ .

## 5. Evaluations

The performance of the Smart-Sleeve is evaluated by using both classical machine learning classifiers and a CNN model. The classical machine learning methods are implemented using Python and Scikit-learn [38] on an Intel Core i7-8700 CPU. The CNN model is implemented using PyTorch [39] on an NVIDIA GeForce 2060 Super GPU. The impacts of the normalization method and the feature set proposed in Section 4 are also evaluated.

### 5.1. Classical Machine Learning Method

**Figure 6:**
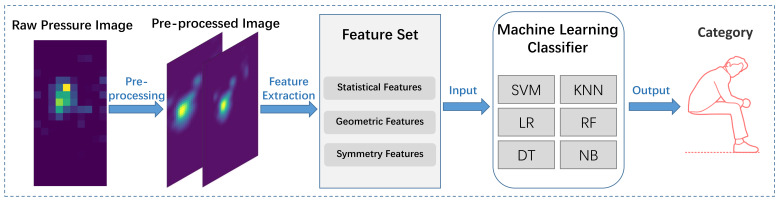
The workflow of classical machine learning method.

The features described above are used to train the following classifiers: support vector machine (SVM), k-nearest neighbor (KNN), logistic regression (LR), random forest (RF), decision tree (DT), and naive Bayes (NB). These algorithms are used widely in IoT devices for HAR [11,40]. The overall workflow is shown in Figure 6. The parameters of these classifiers, such as the number of nearest neighbors (n_neighbors) in KNN and kernel in SVM, are listed in Table 4. The not-specified parameters adopt the default values in Scikit-learn.

### 5.2. Deep Learning Method

**Figure 7:**
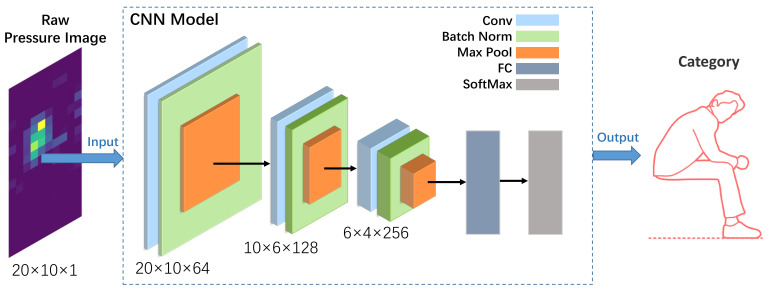
The CNN model. A network consisting of three main blocks is designed to convert the pressure image into a feature space, which is then fed to one dense layer leading to a logistic regressor for recognition of activities.

A deep learning approach (CNN) is also adopted, taking the original samples as the input. As shown in Figure 7, the network consists of three main blocks. Each block is composed of a convolutional layer, a batch normalization layer, a Relu, and a MaxPolling layer. For each convolutional layer, the convolution kernel is 3×3 with a stride of 1 and padding of 1, and the out channels are separately set as 64, 128, and 256. For each Maxpolling layer, the kernel is 2×2 with a stride of 2, and the padding is separately set as (0,1),(1,1) and none. Processed by the three blocks, the input-sized 20×10×1 is converted into the output-sized 3×2×256 . We flatten it into a one-dimensional feature vector and employ a fully connected (FC) layer to classify the activities. We use Adam optimizer at the training stage with the learning rate of $10^{-4}$ and train the network for 30 epochs and the batch size of 40.

### 5.3. Evaluation Metrics

To assess the effectiveness of our methods we use the standard metrics of accuracy, macro precision ( Macro_P ), macro recall ( Macro_R ), and Macro_F1 [41]. Accuracy shows the performance of the model by calculating the number of correct classifications and then dividing it by the number of all samples. $C_{i}$ is used to denote the *i*-th class. The precision of the $C_{i}$ is the ratio of the number of activities classified correctly to the total activities predicted as $C_{i}$ . The recall of the $C_{i}$ is the ratio of the number of activities correctly classified to the number of activities in $C_{i}$ . The Macro_P and the Macro_R are defined as follows.

(3) $$Macro\_P=\frac{1}{m}\times \sum_{i=1}^{m}P_{i}$$

(4) $$Macro\_R=\frac{1}{m}\times \sum_{i=1}^{m}R_{i}$$

Where m is the number of classes, $P_{i}$ denotes the precision of $C_{i}$ , and $R_{i}$ denotes the recall of $C_{i}$ . Macro_F1 is defined in Equation (5), which considers equally important the effectiveness in each class, independently of the relative size of the class.

(5) $$Macro\_F1=\frac{2\times Macro\_P\times Macro\_R}{Macro\_P+Macro\_R}$$

### 5.4. Classical Machine Learning Results

All features are normalized by min–max normalization [42] before classification. Our model is validated based on 10-fold cross-validation (10-fold) and leave-one-subject-out (LOSO). In the 10-fold, we split the data into 10 subsets, where 10% are used for testing and 90% are used for training. The process is repeated 10 times. Finally, we average all the results. In the LOSO scheme, one subject is kept aside at each iteration for testing and the rest of the subjects are used in training. The results are shown in Table 5.

**Figure 8:**
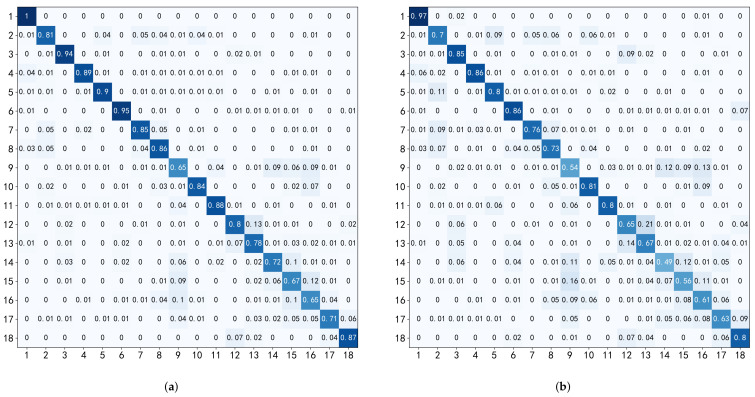
Confusion matrices using the SVM classifier with 10-fold and LOSO validation scheme. The posture categories are represented in Figure 2. (**a**) 10-Fold (SVM). (**b**) LOSO (SVM).

Among all classical classifiers, SVM is the best in all evaluation metrics, which has an accuracy of 82.02% (10-fold) and 72.66% (LOSO). The detailed result is given in Figure 8. Compared to other HAR systems, as illustrated in Table 1, the accuracy of Smart-Sleeve is acceptable. In detail, compared to the first-person camera solution [3], with the same consideration of 18 activities and information about interactions with objects, Smart-Sleeve is not only more accurate but also supports unobtrusive HAR for longer periods of time. Due to the large differences in body size and behavioral habits of different subjects, LOSO exhibits higher errors compared to 10-fold.

Some of the activities, such as activity 3 (think), where most subjects would brace their right arm with their left hand, have better classification accuracies under both SVM and CNN models, due to the fact that they produce significant pressure on the textile surface and have less variation type across subjects. Other activities, such as activity 13 (play with phone), have low classification accuracy. We believe this is caused by different personal habits. For example, we observe that the tilt angle of the right arm to the desktop during playing varies greatly among subjects; some subjects even place their arms directly flat on the desktop. Activity 9 (lean forward at work), activity 14 (write with a hunchback), activity 15 (write with a straight back), and activity 16 (sit with hands on the armrests) all have the forearm placed onto a flat surface, and thus could be easily confused with one another.

To further understand the roles of the normalization method and the new feature set proposed in Section 4, the results with and without the scaling and shift method (A) and the new feature set (B) are compared, listed in Table 6. The 38 spatial features in [33] are used in the situation without the new feature set. All classical machine learning methods listed in Table 4 are used to evaluate based on 10-fold and LOSO.

For each classifier, the result of configuration A is always better than the other. Both the normalization method and the new feature set significantly improve the results. When the number of features is small, the RF classifier shows better results, especially in 10-fold. In general, SVM performs well in a variety of situations.

### 5.5. Deep Learning Results

**Figure 9:**
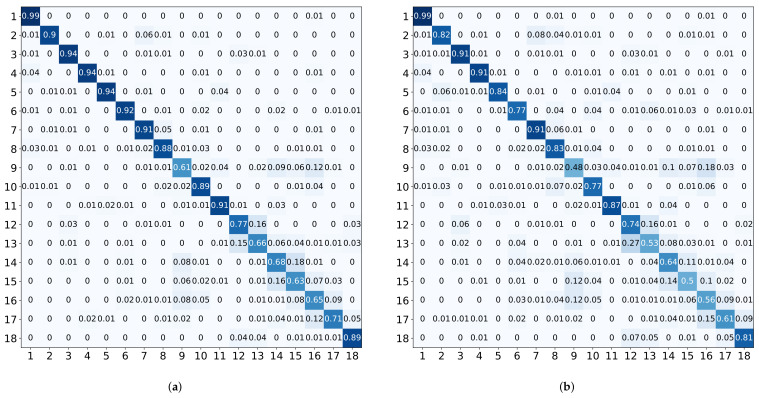
Confusion matrices using CNN. (**a**) 10-Fold (CNN). (**b**) LOSO (CNN).

Table 5 illustrates the deep learning method’s performance, and the average accuracies are 82.30% (10-fold) and 74.84% (LOSO). The CNN model achieves the best result compared to all classical classifiers used in this work. In particular, it performs robustly when faced with samples from subjects not involved in training (LOSO). The detailed result is given in Figure 9. Overall, the variability of classification accuracy across activities is similar to that of SVM. For some activities, such as activity 9 (lean forward at work) and activity 13 (play with phone), the diversity brought by different subjects’ habits and the insufficient number of training samples may lead to lower accuracy. In general, deep learning models require a larger number of training samples.

The CNN framework achieves slightly better results than the SVM classifier without data preprocessing and feature extraction. With sufficient computing power, this will substantially improve our efficiency in developing similar applications. However, our preprocessing and feature extraction are still useful, and methods such as feature selection will help us understand which feature plays a significant role in the model generation process, and thus provide insights into the problems or tasks. More importantly, the workflow of traditional machine learning is better suited to run on inexpensive and low-power IoT devices, supporting real-time HAR applications.

## 6. Conclusions

Human activity recognition is a very challenging research area for the past decades. Our proposed Smart-Sleeve uses pressure information for HAR, which has not been widely studied in the wearable field. Compared to other wearable behavior recognition sensors, the textile pressure sensor array brings good accuracy without compromising wearer comfort. Six classical machine learning classifiers and a convolutional neural network model are used to evaluate the system. Average accuracies of 82.30% (CNN) and 82.02% (SVM) can be achieved in 10-fold cross-validation and 72.66% (SVM) and 74.84% (CNN) in leave-one-subject-out validation. In classical machine learning workflow, to normalize the data differences caused by different body sizes, a new preprocessing method is adopted and a feature set of 100 features, including statistical, geometric, and symmetry features, is proposed. Through experiments, the normalization method and the new feature set are proved to improve classification accuracy significantly, and the proposed CNN model achieves the best result without any data preprocessing and feature extraction. These methods may serve as a reference for similar systems. Our data is also made publicly available.

## 7. Discussions

In the raw pressure image, such as in Figure 4, some of the adjacent points have very different values and the boundary of the pressure area is jagged, which may mean that our sensor density is not high enough. Although upsampling and smoothing are used to attenuate this effect in the data processing, in future work, we plan to further increase the sensor density to provide more accurate activity recognition. In addition, the textile matrix has been tested with simple washing, including the use of household detergent, hot water, and household washing machines, and, subjectively, its sensing performance did not deteriorate significantly. However, more specific indicators, such as sensitivity and range of variation, should be further measured and compared under different washing methods to assess its durability and application scope. For example, if the performance remains good after washing with medical disinfectant, the Smart-Sleeve may be used in hospitals to detect patient activity to assist doctors in tracking the development of disease. We also note that in the results, the recognition accuracies of activities such as playing with phones and working are relatively low, and the accuracy may be further improved by obtaining the usage data of cell phones and computers.

## Figures and Tables

**Table 1:** Comparison of some existing systems for HAR.

Category	Reference	Sensor	Number of Activities	Number of Participants	Classification Technique	Best Accuracy
Environmental	Khan et al., 2022 [4]	vision sensor (Kinect V2)	12	20	Hybrid Deep Learning Model	91%
	Wang et al., 2017 [7]	commercial WiFi device	8	25	Hidden Markov Model	96%
	Sundholm et al., 2014 [19]	textile pressure sensor	10	7	K-nearest Neighbor	90%
Wearable	Pirsiavash et al., 2012 [3]	GoPro camera	18	20	Support Vector Machine	77%
	Jamieson et al., 2021 [13]	accelerometer (ActivPAL)	5	12	Support Vector Machine and Long-Short Term Memory	77%
	Altun et al., 2010 [12]	miniature inertial sensor and magnetometer	19	8	7 kinds of classification techniques ^1^	99%
	Lim et al., 2021 [16]	accelerometer, gyroscope, magnetometer, object, and ambient sensor	18	4	Deep ConvLSTM	91%
	Parzer et al., 2017 [20]	textile pressure sensor	9	6	Support Vector Machine	92%

^1^ Bayesian decision-making, decision tree, least-squares method, k-nearest neighbor, dynamic time warping, support vector machine, and artificial neural networks.

**Table 2:** Advantages and disadvantages of some commonly used sensors for HAR.

Sensor	Advantages	Disadvantages
vision sensors	intuitive	high cost, complex setup and susceptibility to lighting condition and occlusion
wireless devices	device-free and larger coverage	vulnerable to interference from other electromagnetic devices
inertial measurement unit	wearable and motion-related	drift and instability
textile pressure sensors	wearable, contact-related, deformable and stretchable	non-waterproof and instability

**Table 3:** The participants’ demographic and anthropometric characteristics.

Parameters	Mean (±Standard Deviation)	Minimum	Maximum
Age (years)	23.2 (±2.0)	21	28
Height (cm)	173.2 (±9.5)	152	184
Weight (kg)	67.4 (±13.5)	40	85
Arm length (cm)	51.9 (±3.4)	47	59
Forearm length (cm)	25.5 (±1.8)	23	28
Biceps circumference (cm)	29.0 (±4.7)	21	38

**Table 4:** The specified parameters for classical classifiers.

Classifiers	Parameters
SVM	kernel = poly
KNN	n_neighbors = 5
LR	penalty = l2, max_iter = 8000, random_state = 40
RF	class_weight = balanced, criterion = gini, max_features = log2, random_state = 40
DT	random_state = 40
NB	GaussianNB is used, with default parameters

**Table 5:** The results (in %). The classical machine learning classifiers use the results after all the preprocessing methods and features extraction described in Section 4 as input, while the CNN model uses raw samples as input. The best classifier and all best results are bolded.

Classifiers	Accuracy	Macro_P	Macro_R	Macro_F1
		**10-Fold**		
**SVM**	**82.02**	**82.20**	**82.24**	**81.61**
KNN	75.67	76.49	75.95	75.18
LR	76.55	76.33	77.00	75.95
RF	80.20	80.16	80.32	79.51
DT	61.79	62.01	62.19	61.19
NB	61.51	63.08	61.77	60.49
**CNN**	**82.30**	**82.56**	**82.42**	**81.79**
		**LOSO**		
**SVM**	**72.66**	**75.76**	**72.66**	**71.28**
KNN	64.80	67.06	64.80	63.02
LR	70.16	72.64	70.16	68.24
RF	69.72	71.64	69.72	67.62
DT	51.90	52.88	51.90	49.93
NB	57.62	61.34	57.62	54.54
**CNN**	**74.84**	**76.98**	**74.84**	**73.32**

**Table 6:** Evaluations of the normalization method and the feature set (accuracy, in %). All best results are bolded.

Classifiers	with A ^1^ with B (10-Fold)	without A with B (10-Fold)	with A without B (10-Fold)	without A without B (10-Fold)	with A with B (LOSO)	without A with B (LOSO)	with A without B (LOSO)	without A without B (LOSO)
SVM	**82.02**	**78.61**	74.72	69.52	**72.66**	**69.56**	**66.55**	**62.42**
KNN	75.47	75.12	70.40	65.12	64.80	63.81	58.37	54.37
LR	76.55	71.87	68.61	60.99	70.16	66.43	62.94	56.63
RF	80.20	78.25	**75.87**	**73.41**	69.72	67.70	65.60	60.16
DT	61.79	59.44	56.51	56.87	51.90	50.63	48.37	47.02
NB	61.51	59.37	50.83	46.55	57.62	55.20	46.94	42.86

^1^ A is used to represent the normalization method and B is used to represent the new feature set.

## Data Availability

The data presented in this paper are available in a publicly accessible repository (https://github.com/xghgithub/Smart-Sleeve-Dataset (accessed on 26 January 2022)).

## Abbreviations

The following abbreviations are used in this manuscript:

HAR	Human activity recognition
IMU	Inertial measurement units
ADC	Analog to digital converter
SVM	Support vector machine
KNN	k-nearest neighbor
LR	Logistic regression
RF	Random forest
DT	Decision tree
NB	Naive Bayes
CNN	Convolutional neural network
IOT	Internet of Things
LOSO	Leave-one-subject-out
